# Representativeness of reach and effectiveness of proactive tobacco quitline follow**-**up treatment for adults who smoke tobacco products: Secondary analyses of a factorial experiment

**DOI:** 10.18332/tpc/225321

**Published:** 2026-10-15

**Authors:** Danielle E. McCarthy, Julie A. Kirsch, Jesse T. Kaye, Kate Kobinsky, David L. Fraser, Katrina Vickerman, Kristina Mullis, Nayoung Kim, Timothy B. Baker, Michael C. Fiore

**Affiliations:** 1 Center for Tobacco Research and Intervention, University of Wisconsin School of Medicine and Public HealthMadisonUnited States; 2 Department of MedicineDivision of General Internal Medicine, University of Wisconsin School of Medicine and Public HealthMadisonUnited States; 3 Institute on Aging, University of WisconsinMadisonUnited States; 4 Department of Family Medicine and Community HealthUniversity of Wisconsin School of Medicine and Public HealthMadisonUnited States; 5 Center for Wellbeing Research, RVO HealthFort MillUnited States; 6 Department of Health ScienceCollege of Human Environmental Sciences, University of AlabamaTuscaloosaUnited States

**Keywords:** smoking, cessation, quitline, disparities, proactive outreach

## Abstract

**Introduction:**

Tobacco quitlines deliver no-cost evidence-based tobacco use treatment, but many quitline callers continue smoking after a single round of treatment. The aim of this study was to evaluate the representativeness of follow-up quitline treatment reach and effectiveness to inform efforts to enhance quitline re-treatment among adults experiencing socioeconomic disadvantage.

**Methods:**

Secondary analyses used recruitment, self-report, and salivary cotinine data collected from participants in a randomized controlled trial evaluating smoking cessation interventions among previously treated quitline participants with socioeconomic disadvantages recruited in Wisconsin from 7 June 2018 to 25 January 2023. Logistic regression analyses examined differential population representation occurring in re-treatment outreach, screening, enrollment, and biochemically confirmed abstinence.

**Results:**

Proactive outreach was more likely to reach previously treated quitline callers who were aged >45 years (adjusted odds ratio, AOR=1.69–2.33; 95% CI: 1.55–2.63), male (AOR=1.16; 95% CI: 1.08–1.25), Black (AOR=1.18; 95% CI: 1.08–1.30), health-insured (AOR=1.26; 95% CI: 1.11–1.67), less nicotine-dependent (AOR=1.11; 95% CI: 1.02–1.20), co-using alternative tobacco products (AOR=1.46; 95% CI: 1.25–1.70), or living with chronic disease (AOR=1.12; 95% CI: 1.05–1.21). Interest in trial screening was higher among quitline participants who were aged <65 years (AOR=1.45; 95% CI: 1.25–1.89), Black (AOR=2.32; 95% CI: 1.95–2.76), living with chronic disease (AOR=1.34; 95% CI: 1.18–1.53), and had more than a high school education (AOR=1.59; 95% CI: 1.36–1.85). Exclusion criteria differentially excluded Black participants (AOR=1.59; 95% CI: 1.27–2.00) and those with lower nicotine dependence (AOR=1.88; 95% CI: 1.49–2.36). Among trial enrollees, abstinence rates were higher among women (AOR=2.04; 95% CI: 1.37–3.03) and Hispanic (AOR=5.73; 95% CI: 2.68–12.21) participants. Individual differences did not significantly predict treatment engagement or interaction with treatment interventions in predicting abstinence.

**Conclusions:**

Quitline re-treatment outreach to previous users of the quitline may help to close gaps in tobacco treatment among socioeconomically disadvantaged older adults and Black people. Relaxing smoking heaviness and frequency criteria may improve representation of Black people in quitline re-treatment trials. Additional research is needed to guide efforts to improve the representation of groups experiencing disadvantage in quitline trials.

## Introduction

Tobacco quitlines are designed to reduce barriers to tobacco treatment by providing both counseling and nicotine replacement therapy (NRT) remotely, at low or no cost to the people they serve^1^. Quitlines in North America deliver remote evidence-based tobacco cessation counseling and pharmacotherapy to more than 235000 individuals each year^1^, but not all populations benefit equally from quitline services. In 2024, only 0.53% of adults with less than a high school education who smoked, received quitline services in North America^1^. In addition, the vast majority of people who receive quitline counseling and/or quitline-supplied nicotine replacement therapy (NRT) will continue smoking or return to smoking within a few months^1^. Re-engaging people who do not achieve lasting abstinence after an initial round of treatment^2^ may be an efficient way to enhance the effectiveness of quitline services, similar to adaptive, stepped care approaches^3^. Targeting re-treatment outreach to people who experience socioeconomic disadvantage may also help to reduce tobacco-driven health disparities by focusing quitline follow-up resources on populations that are most affected by tobacco use and have the greatest difficulty quitting^4^.

Clinical trials focused on quitline re-treatment strategies need to be designed in ways that enhance their generalizability to populations at greatest risk of not responding fully to a single round of quitline treatment. Designing recruitment strategies with representativeness in mind may enhance such trials and help to identify subpopulations that may need alternative or additional approaches to recruitment^5-11^. People from minoritized groups and disadvantaged backgrounds tend to be underrepresented in clinical research^6,7,10^, including research on tobacco use interventions^8,9,11^. Given this, it is important to assess the representativeness of enrollment in quitline retreatment trials to identify whom we may be missing and ways to enhance inclusion in future trials.

The present study is a secondary analysis of data from a recent randomized controlled trial of proactive quitline re-treatment outreach and interventions for people who did not achieve lasting abstinence from smoking after standard quitline treatment^12^. The parent clinical trial assessed ways to enhance quitline effectiveness among people with socioeconomic disadvantage through targeted proactive re-treatment, with or without randomly assigned service enhancements^12^. The clinical trial included 1316 Wisconsin Tobacco Quit Line (WTQL) service participants with one or more indicators of socioeconomic disadvantage (i.e. Medicaid eligibility, no insurance, and/or no more than a high school education or a graduate equivalent degree or GED) and provided at least one additional round of standard quitline coaching and NRT^12^. The present analyses assessed the representativeness of the retained sample at key steps in the treatment process, from initial outreach to confirmation of abstinence 6 months after a target quit date. The aim of this secondary analysis was to identify gaps in the representativeness of quitline re-treatment reach and effectiveness and inform strategies to address such gaps.

## Methods

### Parent trial design

The present study is a secondary analysis of data collected from WTQL participants recruited between 7 June 2018 and 25 January 2023 for a 2×2×2×2 factorial experiment that evaluated the main and interactive effects of 4 intervention components: 1) Quitline counseling (1 call vs 4 calls); 2) NRT (2 weeks of nicotine patch vs 4 weeks of nicotine patch with nicotine lozenge); 3) SmokefreeTXT (enrollment information for the National Cancer Institute text messaging program vs no information); 4) Incentives for treatment engagement ($30 for each counseling call completed and staying in SmokefreeTXT for at least 6 weeks post-quit-date vs no incentives). All enrolled participants set a quit date within 2 weeks of enrollment and consented to follow-up assessments at 12 and 26 weeks following the quit date. Additional details are available via ClinicalTrials.gov (NCT03538938) and the primary outcome publication^12^.

### Participants

For participants to be eligible for recruitment outreach, they needed to satisfy the following criteria: aged ≥18 years, completed at least one counseling call in English with the WTQL in the past 3–6 months, reside in Wisconsin, not share a phone number with someone already enrolled in the trial, and have at least one indicator of socioeconomic disadvantage (Medicaid insurance, no health insurance, and/or no more than a high school education or GED).

Participants in the clinical trial had to meet additional inclusion criteria at recruitment: smoking at least for 6 of the past 7 days, smoking ≥5 cigarettes at least 1 of these smoking days, and being willing to set a quit date within 2 weeks. Female participants had to report that they were not pregnant, breastfeeding, or planning to become pregnant in the next 6 months. To receive NRT, participants had to meet standing WTQL criteria. Enrollment in SmokefreeTXT required controlled access to a mobile device and consent to join the program.

### Procedures

The study proactively recruited adult participants 3–6 months after they enrolled in an index round of WTQL counseling (with or without NRT). Quit Line personnel trained in the study protocol made up to 5 attempts to reach prospective participants to assess their current smoking status using a single item: ‘Have you smoked at all, even a puff, in the last 7 days?’. Those reporting no smoking were congratulated and offered standard WTQL support. Those who reported any smoking in the past 7 days were given a brief introduction to the clinical trial and asked if they assented to eligibility screening. Those who passed the screening were asked to provide oral consent for participation. Those who declined trial participation were offered standard WTQL services. Consented trial participants completed a baseline phone survey, were randomized to 1 of 16 conditions, and completed a counseling call. This typically occurred in a single phone call. Participants were then mailed a welcome packet tailored to their randomized condition. Those eligible for medication were mailed NRT with use instructions.

Phone assessments occurred at 12 and 26 weeks post-quit and assessed use of tobacco products and smoking cessation treatments. Participants reporting no smoking in the past 7 days at the follow-up at 26 weeks, were asked to provide a saliva sample for cotinine testing. A sample collection kit was mailed to eligible participants, with a stamped return envelope. Researchers called participants to encourage sample return.

All recruitment, enrollment, and treatment activities were completed by WTQL personnel, while the post-quit assessments at 12 and 26 weeks were completed by research personnel at the University of Wisconsin. The Health Sciences Institutional Review Board (IRB) at the University of Wisconsin approved all study procedures.

### Measures

Participant characteristics were assessed primarily with existing WTQL items gathered at registration. These items follow the Minimal Data Set (MDS) guidelines published by the North American Quitline Consortium^13^. Items analyzed for this study include: region of Wisconsin (based on 3-digit zip code and coded as Northeast [reference group], Southeast, Southern, Western, or missing); self-reported sex (female [reference group], male, or missing); age (18–44 years [reference group], 45–64 years, ≥65 years, or missing); race (Black/African American, White [reference group], Other, or missing); ethnicity (Hispanic or Latino, Not Hispanic/Latino [reference group] or missing); health insurance type (Medicaid, Medicare, Commercial or other non-Medicaid or -Medicare insurance [reference group], uninsured, or missing); education level (lower than grade 12/GED, grade 12/GED, some college or higher [reference group], or missing); types of tobacco product used (cigarettes only [reference group], cigar, pipe, or smokeless tobacco; or hookah, e-cigarettes, or other alternative nicotine device, or missing); time to first cigarette from the Fagerström test for nicotine dependence^14^ (within 30 min of waking, after 30 min of waking [reference group], or missing); and chronic health conditions (yes if reported asthma, chronic obstructive pulmonary disease, coronary artery disease, and/or diabetes, no if reported none of these conditions [reference group], or missing). All these variables were examined as predictors of inclusion in the trial to assess the representativeness of the sample retained at each step in the recruitment, enrollment, treatment, and outcome assessment process. None was considered to be a confounder.

Participant demographics were reviewed at enrollment, and additional characteristics were assessed in clinical trial participants. These included annual household income from all sources and the national percentile of the Area Deprivation Index (ADI, Version 3.0)^15,16^ for the participant’s address at enrollment. The ADI ranks census blocks based on Census and American Community Survey measures of education, employment, housing, and poverty^15^. Higher scores on the ADI indicate greater neighborhood socioeconomic disadvantage.

Outcome variables included binary indicators of reaching/completing each phase in the requitting process: outreach (e.g. 1 if reached, 0 if not reached), screening, eligibility, enrollment, treatment engagement (i.e. completing at least 1 counseling call, receiving NRT, and using NRT), and reporting abstaining from smoking for at least 7 days at the post-quit-date follow-up at 12 weeks and 26 weeks. At the follow-up at 26 weeks, self-reported abstinence was confirmed by salivary cotinine (cutoff 10 ng/mL: Forensic Fluids Laboratories, Kalamazoo, MI). Additional outcomes included specific reasons for exclusion at initial outreach and eligibility screening, and binary indicators of completion of the follow-up at 12 weeks and 26 weeks, and of submitting a saliva sample among those reporting abstinence at the follow-up at 26 weeks. For all outcomes, missing cases were treated as not having the outcome (e.g. not reached, not screened, not abstinent). Some outcomes occurred at very low frequencies and were not analyzed, including exclusion due to pregnancy, breastfeeding, or planning to become pregnant (n=18) and not completing at least one counseling call (n=81).

### Statistical analysis

Descriptive statistics were used to characterize the full sample of people eligible for trial outreach, for those enrolled in the trial, and for those not enrolled in the trial. Descriptive analyses also captured retention and loss at each step in trial flow to inform the development of coding schemes for predictors. Variables with many missing cases were coded in a separate missing category rather than excluded because differential rates of missingness across items suggest that these data are unlikely to be missing at random and instead may reflect participant attitudes that are non-ignorable. We elected not to use multiple imputation to address missing data because the missingness is potentially informative. In primary analyses, the ADI percentile was treated as a continuous variable. Sensitivity analyses used a dummy indicator of ADI (≥ median of 85) to assess whether this altered the pattern of results.

Logistic regression analyses were conducted for each outcome with sufficient variability for analyses of representativeness. We conducted both bivariate models with a single predictor and multiple logistic regression models with Benjamini-Hochberg corrections^17^ to control the false discovery rate at alpha=0.05. Corrected multiple logistic regression analyses were considered primary and are summarized in Supplementary file Figure S1. We did not have *a priori* hypotheses regarding interactions between patient characteristics and intervention components, but conducted exploratory analyses using hierarchical multiple logistic regression to examine such effects. Analyses were conducted in IBM SPSS Statistics for Windows, version 28.0.0.0 (IBM Corp., Armonk, NY).

## Results

### Descriptive results

Flow through study procedures, from initial outreach to confirming abstinence 6 months after a target quit date, is shown in Supplementary file Figure S1. A total of 14125 people met the prerequisites for proactive outreach from the quitline. Characteristics of this sample are presented in Table 1. As shown in Table 1, participants were drawn from all regions of Wisconsin, with the greatest representation in the most densely populated region of the state, the Southeast (50.4%). The majority of participants (62.2%) were aged 45–64 years, with fewer <45 years (23.3%) or >65 years (14.2%). More than half of the participants were female (57.8%), while 41.8% were male. Four percent of participants identified as Hispanic or Latino. In terms of race, 26.3% identified as Black or African American, 64.4% as White, 6.5% as another race or multiple races. The majority of participants were covered by Medicaid insurance (56.1%), while 19.4% were covered by Medicare, 12.5% were covered by commercial or other insurance, and 9.9% reported having no insurance. In collinearity checks, correlations among predictor variables were modest (all r<0.37).

**Table 1:** Characteristics reported at Wisconsin Tobacco Quit Line registration by participants who were eligible for recruitment between 7 June 2018 and 25 January 2023 in the parent factorial experiment of this secondary analysis study based on data reported at quitline registration 3–6 months prior to recruitment (N=14125)

Characteristics	Category	Enrolled (N=1316)	Not enrolled (N=12809)	Total(N=14125)	p^*^
**Region of Wisconsin**	Northeast	209 (15.9)	2221 (17.3)	2430 (17.2)	<0.001
	Southeast	663 (50.4)	5791 (45.2)	6454 (45.7)	
	Southern	255 (19.4)	2435 (19.0)	2690 (19.0)	
	Western	183 (13.9)	2321 (18.1)	2504 (17.7)	
	Missing	6 (0.5)	41 (0.3)	47 (0.3)	
**Age** (years)	18–44	306 (23.3)	4191 (32.7)	4497 (31.8)	<0.001
	45–64	818 (62.2)	6586 (51.4)	7404 (52.4)	
	65–90	187 (14.2)	2013 (15.7)	2200 (15.6)	
	Missing	5 (0.4)	19 (0.1)	24 (0.2)	
**Sex**	Female	760 (57.8)	7533 (58.8)	8293 (58.7)	0.227
	Male	550 (41.8)	5247 (41.0)	5797 (41.0)	
	Missing	6 (0.5)	29 (0.2)	35 (0.2)	
**Ethnicity**	Not Hispanic/Latino	1245 (94.6)	11907 (93.0)	13152 (93.1)	0.006^§^
	Hispanic or Latino	53 (4.0)	536 (4.2)	589 (4.2)	
	Missing	18 (1.4)	366 (2.9)	384 (2.7)	
**Race**	Black/African American	346 (26.3)	2516 (19.6)	2862 (20.3)	<0.001
	White	848 (64.4)	9241 (72.1)	10089 (71.4)	
	Other	85 (6.5)	749 (5.8)	834 (5.9)	
	Missing	37 (2.8)	303 (2.4)	340 (2.4)	
**Health insurance type**	Medicaid	738 (56.1)	6378 (49.8)	7116 (50.4)	<0.001
	Medicare	255 (19.4)	2519 (19.7)	2774 (19.6)	
	Commercial or other	164 (12.5)	1837 (14.3)	2001 (14.2)	
	Uninsured	130 (9.9)	1757 (13.7)	1887 (13.4)	
	Missing	29 (2.2)	318 (2.5)	347 (2.5)	
**Education**	Lower than grade 12/GED	290 (22.0)	2277 (17.8)	2567 (18.2)	<0.001
	Grade 12/GED	532 (40.4)	6653 (51.9)	7185 (50.9)	
	Some college or higher	463 (35.2)	3636 (28.4)	4099 (29.0)	
	Missing	31 (2.4)	243 (1.9)	274 (1.9)	
**Chronic condition**	No	631 (47.9)	7525 (58.7)	8156 (57.7)	<0.001
	Yes	680 (51.7)	5265 (41.1)	5945 (42.1)	
	Missing	5 (0.4)	19 (0.1)	24 (0.2)	
**Tobacco product use**	Cigarettes only	1127 (85.6)	11166 (87.2)	12293 (87.0)	0.122
	Cigar, pipe, or smokeless tobacco	110 (8.4)	991 (7.7)	1101 (7.8)	
	Hookah, e-cigarettes, or other	74 (5.6)	633 (4.9)	707 (5.0)	
	Missing	5 (0.4)	19 (0.1)	24 (0.2)	
**Time to first cigarette**	Within 30 min	1118 (85.0)	10057 (78.5)	11175 (79.1)	<0.001
	After 30 min	188 (14.3)	2583 (20.2)	2771 (19.6)	
	Missing	10 (0.8)	169 (1.3)	179 (1.3)	

* Chi-squared tests by study enrollment status.

§ p=0.704 when cases missing ethnicity are excluded.

### Outreach

Phone contact was achieved for 6019 (42.6%) of those called and varied across participants. After Benjamini-Hochberg correction in multiple logistic regression analyses, rates of reaching people were higher for: middle-aged (45–64 years: AOR=1.69; 95% CI: 1.55–1.83) or older (≥65 years: AOR=2.33; 95% CI: 2.07–2.63) people than in those <45 years; men (AOR=1.16; 95% CI: 1.08–1.25 vs women); Black people (AOR=1.18; 95% CI: 1.08–1.30 vs White people); those with Medicare (AOR=1.19; 95% CI: 1.05–1.34 vs commercial insurance); those who used electronic or other alternative nicotine delivery systems (AOR=1.46; 95% CI: 1.25–1.70 vs those who did not); and those who reported having at least one of the four chronic health conditions assessed (AOR=1.12; 95% CI: 1.05–1.21 vs no chronic condition). Rates of reaching people via outreach were lower for: people who did not report their ethnicity at their recent quitline registration (AOR=0.69; 95% CI: 0.52–0.92 vs non-Hispanic or Latino people); those without health insurance (AOR=0.79; 95% CI: 0.68–0.90 vs commercial insurance); and those who smoked within 30 min of waking (AOR=0.90; 95% CI: 0.83–0.98 vs after >30 min). Full results from bivariate and multiple logistic regression analyses are shown in Supplementary file Table S1.

A total of 1635 (11.6% of the full sample) individuals reached by phone were excluded due to reporting not smoking at the re-treatment outreach call. Longer latency to smoke after waking at initial quitline registration predicted greater odds of reporting abstinence at outreach (AOR=1.52; 95% CI: 1.32–1.74). No other predictors were significantly related to abstinence at outreach after correction (Supplementary file Table S2).

### Screening

Of those reporting smoking at outreach, 2375 (16.8% of the full sample) completed trial screening. Screening completion rates were significantly higher among participants who identified as Black (AOR=2.32; 95% CI: 1.95–2.76 vs White) and those who had one of the assessed chronic health conditions (AOR=1.34; 95% CI: 1.18–1.53 vs no chronic condition). Screening completion rates were lower among: participants who were >65 years (AOR=0.65; 95% CI: 0.53–0.80 vs those <45 years); those who did not complete high school (AOR=0.61; 95% CI: 0.50–0.74) and those who completed high school or a GED but did not attend college (AOR=0.64; 95% CI: 0.54–0.75) versus those who had some college; and those not reporting their ethnicity at registration (AOR=0.47; 95% CI: 0.27–0.82 vs non-Hispanic or Latino participants). Full results are given in Supplementary file Table S3.

### Eligibility

A total of 1744 (12.3% of the full sample) met trial eligibility criteria (Supplementary file Table S4). Eligibility likelihood was elevated in those who smoked within 30 min of waking (AOR=1.88; 95% CI: 1.49–2.36 vs those who waited >30 min). People who identified as Black (AOR=0.63; 95% CI: 0.50–0.79 vs those who identify as White) and people who did not report their ethnicity (AOR=0.25; 95% CI: 0.11–0.56 vs non-Hispanic/Latino participants) passed screening at lower rates. Black individuals were excluded for not smoking enough in the past 7 days at twice the rate of White individuals (AOR=2.03; 95% CI: 1.57–2.62). Those who did not report their ethnicity were nearly 4 times more likely to be ineligible due to insufficient smoking (AOR=3.92; 95% CI: 1.72–8.97). See Supplementary file Table S5 for full results. Unwillingness to set a quit date did not differ significantly across populations after correction (Supplementary file Table S6).

### Enrollment

A total of 1316 (9.3% of the full sample) participants consented and enrolled in the parent trial. Reasons for not enrolling are summarized in Supplementary file Figure S1, and the characteristics of those enrolled versus not enrolled are reported in Table 1, along with the p-values for chi-squared tests. In multiple regression analyses with correction (Supplementary file Table S7), people with a high school education or GED (AOR=0.60; 95% CI: 0.45–0.79 vs those with at least some college) and those who did not report their ethnicity (AOR=0.12; 95% CI: 0.03–0.45 vs non-Hispanic or Latino participants) were less likely to enroll than their counterparts.

### Treatment engagement

Among 1316 trial enrollees, 1235 (93.8%) completed at least one counseling call with a Quit Coach. Because this rate was so high, we did not analyze differences in completion of counseling calls across participant groups. We did not analyze the number of calls completed, as this was a manipulated experiment, and roughly half of the participants had access to only one proactive counseling call. Rates of NRT use were more variable across participants. A total of 1060 (80.5%) enrollees were eligible for and agreed to use NRT. At the follow-up phone survey at 12 weeks, 966 (73.4%) of enrollees reported they had used NRT since enrolling. Rates of any use of NRT at the follow-up at 26 weeks, were lower with 555 (42.2%) of enrollees reporting use since the last assessment. Rates of receiving NRT and using NRT at 12 weeks did not vary significantly across enrollee populations, but any use of NRT at 26 weeks was reported more frequently by participants aged ≥65 years (AOR=2.42; 95% CI: 1.51–3.86 vs those <45 years) and by Black participants (AOR=1.56; 95% CI: 1.14–2.14 vs White participants). See Supplementary file Tables S8-S10 for full results. Treating ADI as binary (≥ the median ADI of 85 vs <85) did not alter the pattern of results compared with analyses that treated ADI as continuous.

Exploratory analyses of NRT engagement did not detect differences in receiving or reporting using NRT as a function of patient characteristics and randomly assigned NRT condition. We detected no significant interactions between randomly assigned NRT condition (4 weeks of nicotine patch and lozenge vs 2 weeks of nicotine patch) and patient characteristics following correction.

### Abstinence

In analyses in which missing=smoking, 25.5% of all enrollees reported no smoking in the past 7 days at the follow-up at 12 weeks, and 12.3% of enrollees provided salivary biochemical confirmation of abstinence (<10 ng/mL cotinine) at the follow-up at 26 weeks. Enrolled participants who identified as Hispanic or Latino were 2.89 times more likely (95% CI: 1.49–5.63) to report abstinence at 12 weeks (Supplementary file Table S10) and 5.73 (95% CI: 2.68–12.21) times more likely to have biochemically verified abstinence at 26 weeks (Supplementary file: Table S11 and Figure S1) than were non-Hispanic/Latino participants. Men were less likely (AOR=0.49; 95% CI: 0.33–0.73) than women to have confirmed abstinence at the follow-up at 26 weeks. No other differences survived correction (Supplementary file Tables S11 and S12). Treating ADI as binary (≥ the median ADI of 85 vs <85) did not alter the pattern of results compared with analyses that treated ADI as continuous. Exploratory moderation analyses with correction did not detect any significant interactions between patient characteristics and the main effects of the 4 intervention components. Hispanic ethnicity and male sex remained significant with interactions when treatment conditions were included in models. No predictors of abstinence were significantly related to follow-up completion or saliva sample returns in Benjamini-Hochberg-corrected multiple logistic regression analyses.

## Discussion

This research found evidence of differential inclusion of different populations of individuals with socioeconomic disadvantage across multiple phases of quitline re-treatment flow: i.e. across quitline outreach, screening, enrollment, engagement, and abstinence. Importantly, the populations that were differentially included varied across phases in the treatment process. In addition to differential inclusion, analyses identified patient characteristics predictive of successful smoking cessation with quitline re-treatment, specifically female sex and Hispanic or Latino ethnicity. Such phase-related patterns of inclusion and success may provide useful information for future efforts to achieve more inclusive and effective methods across all phases of quitline re-treatment trials.

Proactive outreach amongst those still smoking after standard quitline treatment was more effective in reaching those aged >45 years, men, Black people, people with Medicare, people with chronic health conditions, those with insurance, and those with markers of lower nicotine dependence. Further, people with lower level of education and those aged >64 years who were still smoking at outreach, were less likely to agree to complete screening for the clinical trial. Smoking heaviness and frequency criteria for inclusion in the clinical trial disproportionately excluded Black participants and those who were less dependent on cigarettes. Also, not responding to a quitline registration question on ethnicity predicted lower outreach success, screening completion, trial eligibility, and trial enrollment. In contrast, among those enrolled in the trial, Hispanic or Latino ethnicity was associated with markedly higher rates of confirmed abstinence 26 weeks after a target quit date.

This research aligns with prior research in suggesting that proactive treatment outreach calls can promote re-treatment initiation^2^ and can effectively recruit some priority populations, such as Black individuals^5,18-21^. The current results also align with evidence that excluding individuals who smoke less heavily reduces the inclusion of Black individuals in tobacco treatment trials^5^, consistent with epidemiological data showing reduced cigarette consumption in Black and Hispanic or Latino populations^22^.

It is difficult to interpret the finding that missing ethnicity data predicted lower odds of answering quitline outreach calls and completing screening. These associations were robust to adjustment for other sociodemographic characteristics (including missing race, insurance, or education). Future research is needed to replicate this finding and explore reasons for non-disclosure of sociodemographic information, and ethnicity in particular. Although past research suggests that rates of missing race and ethnicity in health insurance registration data are higher among people from minoritized backgrounds, we cannot be sure this is the case in the present study^23^.

The present study found that limited education may be a barrier to inclusion in quitline trials as it was related to lower rates of trial screening and enrollment. This is notable given that this sample was selected for disadvantage (limited education, Medicaid eligibility, and/or no health insurance). The robustness of the relation between lower level of education and declining trial participation, even in a sample with a restricted range, indicates the importance of this factor in reducing representativeness in clinical trials and adds to prior literature suggesting that cancer trial participation is significantly lower among people with lower level of education and lower income, potentially due to differing perceptions of trial benefits and costs^24^.

The finding that participants aged >65 years were less likely to agree to the quitline re-treatment trial screening is consistent with evidence that older individuals have reduced quitting motivation^25,26^, but should be interpreted cautiously pending replication. Past research suggests that perceptions of the risks of smoking and benefits of quitting differ between those ≥60 years versus <60 years^27^. Future research could explore motivational strategies (e.g. messages focused on common and salient concerns about future health problems^28,29^) that increase treatment and research participation in older individuals who smoke.

Quitline participants with chronic conditions (asthma, chronic obstructive pulmonary disease, coronary artery disease, or diabetes) were reached and screened for re-treatment trial participation at higher rates than those without these conditions. This differs from recent US population data, which do not show differences in interest in quitting smoking, past year quit attempts, or recent successful cessation as a function of chronic disease status^25^.

Tobacco use history also predicted engagement outcomes. Participants who used alternative tobacco products such as e-cigarettes were more likely to be reached by the quitline via proactive re-treatment outreach. The use of alternative products may reflect ongoing efforts to reduce combusted cigarette smoking^30^ and pre-existing high levels of interest in tobacco cessation. Use of alternative products was not significantly associated with abstinence after the initial round of quitline treatment or after re-treatment in the randomized controlled trial, however.

Shorter latency to the first cigarette of the day, a marker of greater nicotine dependence^31^, was also associated with engagement outcomes. Those who smoked within 30 min of waking were less likely to be reached by the quitline for re-treatment but were more likely to be eligible for the re-treatment trial (perhaps because they were not excluded for insufficient smoking).

### Limitations

Certain factors limit generalization from these results. First, the nature of the population, treatments, and outreach methods may limit generalization beyond Wisconsin quitline participants. For instance, the initial, low-intensity quitline treatment that prospective trial participants received (one coaching call and 2 weeks of a single NRT medication, plus web-based and mailed materials) likely affected the types of individuals available for re-treatment; these findings may not generalize to quitline programs that offer more coaching and NRT. Also, some treatment elements varied over the study period: e.g. the standard text messaging service offered by the quitline was not available for much of the study, and an enhanced treatment program for American Indian populations was added during the study. Also, analyses were limited to variables collected routinely by the quitline, some variables (e.g. race, ethnicity, insurance type) were often missing, and residual confounding may have affected results in adjusted models. Further, race and ethnicity were assessed using broad categories, with limited representation of non-Black minoritized populations. Finally, self-report data are subject to biases, and rates of returning saliva samples for biochemical verification of abstinence at follow-up at 26 weeks were low, which may have contributed to misclassification of abstinence status.

## Conclusions

The current secondary analyses extend the literature on the representativeness of quitline re-treatment and identify factors associated with quitline re-treatment trial reach, engagement, and effectiveness. Results suggest that proactive efforts to re-engage quitline participants in services may help reduce gaps in tobacco treatment use among older adults, Black people, and those with chronic health conditions. Differential screening and enrollment rates suggest that the representativeness of randomized clinical trials could be enhanced by relaxing smoking heaviness and frequency criteria for entry into treatment trials. Additional efforts may be needed to re-engage people without insurance, those who are older than 65 years, and those who are highly nicotine dependent in quitline re-treatment. Additional efforts are also needed to ensure people with lower than a high school education are well represented in quitline trials.

## Abbreviations

ADI: Area Deprivation Index
AOR: adjusted odds ratio
CAD: Coronary Artery Disease
CI: confidence interval
COPD: Chronic Obstructive Pulmonary Disease
GED: Graduate Equivalent Degree
NRT: nicotine replacement therapy
RB: Institutional Review Board
WTQL: Wisconsin Tobacco Quit Line
