# Supplementary material for: Representativeness of reach and effectiveness of proactive tobacco quitline follow-up treatment for adults who smoke tobacco products: Secondary analyses of a factorial experiment
Source: Tob Prev Cessat. 2026 Oct 15;12:10.18332/tpc/225321. doi: 10.18332/tpc/225321 (PMC13687672; doi:10.18332/tpc/225321)

TOBACCO PREVENTION AND CESSATION

Supplementary file

© 2026 McCarthy D.E. et al.

DOI:
10.18332/tpc/225321

The content has been provided by the author(s) and has not been reviewed, verified, or endorsed by European Publishing. It may not have undergone peer review. The views, opinions, and recommendations expressed are solely those of the author(s) and do not necessarily reflect the position of European Publishing. European Publishing accepts no responsibility or liability for any consequences arising from the use of, or reliance on, this content.

SUPPLEMENTARY MATERIALS

Supplemental Tables 1-12 display results of logistic regression analyses predicted outcomes at each step in the outreach, screening, enrollment, treatment, and cessation processes assessed. Unadjusted analyses were bivariate analyses that examined relations between each predictor and outcome without including other predictors. Adjusted analyses were multiple logistic regression analyses that included all predictors simultaneously. Each table presents unadjusted odds ratios (OR) for bivariate relations between each predictor and outcome and adjusted odds ratios (aOR) from multiple logistic regression analyses, along with associated 95% confidence intervals (95%CI), *p* values, and risk differences (difference in the estimated probabilities of the outcome when the predictor is present versus absent in the adjusted models). In abstinence analyses at 12- and 26- weeks post-target-quit-date, additional analyses were conducted to include parent trial intervention component main effects, interactions among these components, and interactions between each component main effect and each individual difference predictor. None of the interactions between intervention components and individual differences were significant at Benjamini-Hochberg-corrected *p* values. As such, these additional analyses are not presented in the supplemental tables.

Table Index

Table S1. Logistic regression analyses of reaching potentially eligible participants on the phone.

Table S2. Logistic regression analyses of 7-day point-prevalence abstinence of WTQL clients who were reached for study recruitment.

Table S3. Logistic regression analyses of completing screening with potentially eligible participants on the phone.

Table S4. Logistic regression analyses of meeting eligibility criteria at the study phone screen.

Table S5. Logistic regression analyses of exclusion due to insufficient smoking out of all clients who were screened and not excluded due to pregnancy.

Table S6. Logistic regression analyses of exclusion due to unwillingness to set a quit date within the next 2 weeks out of all clients who met other eligibility criteria.

Table S7. Logistic regression analyses of trial enrollment among all those who met screening criteria.

Table S8. Logistic regression analyses of receiving NRT among all enrolled and randomized trial participants.

Table S9. Logistic regression analyses of any self-reported use of NRT at the 12-week post-quit follow-up call among participants who completed the 12-week follow-up.

Table S10. Logistic regression analyses of any self-reported use of NRT at the 26-week post-quit follow-up call among participants who completed the 26-week follow-up.

Table S11. Logistic regression analyses of self-reported abstinence from smoking in the past 7 days at the 12-week post-quit follow-up call among all randomized enrollees.

Table S12. Logistic regression analyses of salivary cotinine-confirmed abstinence from smoking in the past 7 days at the 26-week post-quit follow-up call among all randomized enrollees.

Supplementary Table S1. Unadjusted (bivariate) and adjusted (multiple) logistic regression analyses of reaching potentially eligible participants on the phone (6,019 of 14,125, or 42.6%, of participants were reached, n in this analysis is 13,935 due to missing data).

	Unadjusted				*Adjusted*				
Predictor	*OR*	*Low 95%CI*	*High 95%CI*	*p*	*aOR*	*Low 95%CI*	*High 95%CI*	*p*	*Risk Difference*
Wisconsin region									
Northeast (Ref)	1.00	-	-	-	1.00	-	-	-	-
Southeast	1.09	0.99	1.19	.088	1.00	0.90	1.10	.958	2.11%
South	1.05	0.94	1.18	.371	1.07	0.96	1.20	.239	0.40%
West	0.94	0.84	1.05	.260	0.97	0.86	1.09	.555	-2.87%
Age									
18 to 44 (Ref)	1.00	-	-	-	1.00	-	-	-	-
45 to 64	1.74	1.61	1.88	<.001	1.69	1.55	1.83	<.001*	5.41%
65 to 90	2.60	2.34	2.88	<.001	2.33	2.07	2.63	<.001*	14.86%
Sex									
Female (Ref)	1.00	-	-	-	1.00	-	-	-	
Male	1.13	1.06	1.21	<.001	1.16	1.08	1.25	<.001*	3.05%
Ethnicity									
Not Hispanic or Latino (Ref)	1.00	-	-	-	1.00	-	-	-	-
Hispanic or Latino	1.02	0.86	1.20	.842	1.16	0.97	1.39	.110	0.95%
Missing	0.84	0.68	1.04	.104	0.69	0.52	0.92	.012*	-4.80%
Race									
White (Ref)	1.00	-	-	-	1.00	-	-	-	-
Black	1.17	1.07	1.27	<.001	1.18	1.08	1.30	<.001*	3.83%
Other Race	1.00	0.86	1.15	.946	1.07	0.91	1.25	.426	-1.15%
Missing	1.02	0.82	1.27	.853	1.16	0.85	1.58	.352	0.33%
Health insurance type									
Commercial (Ref)	1.00	-	-	-	1.00	-	-	-	-
Medicaid	1.00	0.90	1.10	.170	1.01	0.91	1.13	.856	-2.07%
Medicare	1.54	1.37	1.73	<.001	1.19	1.05	1.34	.008*	11.94%
Uninsured	0.74	0.65	0.85	<.001	0.79	0.68	0.90	<.001*	-9.32%
Missing	0.80	0.63	1.01	.063	0.75	0.59	0.97	.026	-7.68%
Education									
Some college or more (Ref)	1.00	-	-	-	1.00	-	-	-	-
Less than grade 12/GED	1.34	1.22	1.48	<.001	1.12	1.00	1.25	.042	5.41%
Grade 12 or GED	1.12	1.03	1.21	.005	0.98	0.90	1.07	.654	0.05%
Missing	1.19	0.93	1.53	.302	1.33	0.92	1.91	.126	1.01%
Has chronic condition	1.32	1.23	1.41	<.001	1.12	1.05	1.21	.001*	6.67%
Other tobacco products									
Uses cigar, pipe, or smokeless tobacco	1.09	0.96	1.23	.200	1.15	1.01	1.31	.034	2.40%
Uses other tobacco (e.g., hookah, e-cigarettes)	1.32	1.15	1.51	<.001	1.46	1.25	1.70	<.001*	8.56%
Smokes within 30 minutes of waking	0.90	0.82	0.97	.010	0.90	0.83	0.98	.018*	-2.64%

Hosmer-Lemeshow goodness-of-fit Chi Square(df=8)=12.83, p=0.118 for the adjusted model. OR=unadjusted odds ratio; aOR=adjusted odds ratio, 95%CI=95% confidence interval; Risk difference=difference in estimated outcome probability in adjusted model; Ref=reference group; * *p* value<Benjamini-Hochberg critical value.

Supplementary Table S2. Unadjusted (bivariate) and adjusted (multiple) logistic regression analyses of 7-day point-prevalence abstinence of WTQL clients who were reached for study recruitment (1,635 of 6,019, or 27.2% of participants reached reported no smoking in the past 7 days).

	Unadjusted				*Adjusted*				
Predictor	*OR*	*Low 95%CI*	*High 95%CI*	*p*	*aOR*	*Low 95%CI*	*High 95%CI*	*p*	*Risk Difference*
Wisconsin region									
Northeast (Ref)	1.00	-	-	-	1.00	-	-	-	-
Southeast	0.95	0.81	1.12	.517	0.98	0.83	1.17	.854	-3.11%
South	1.12	0.93	1.35	.450	1.11	0.92	1.35	.285	1.71%
West	1.23	1.01	1.49	.035	1.20	0.98	1.45	.076	3.94%
Age									
18 to 44 (Ref)	1.00	-	-	-	1.00	-	-	-	-
45 to 64	0.80	0.70	0.91	.001	0.84	0.73	0.97	.021	-4.12%
65 to 90	0.97	0.82	1.15	.729	1.00	0.83	1.22	.969	1.97%
Sex									
Female (Ref)	1.00	-	-	-	1.00	-	-	-	-
Male	1.19	1.06	1.33	.003	1.14	1.01	1.28	.035	3.37%
Ethnicity									
Not Hispanic or Latino (Ref)	1.00	-	-	-	1.00	-	-	-	-
Hispanic or Latino	0.79	0.58	1.06	.116	0.81	0.59	1.12	.204	-4.02%
Missing	1.05	0.73	1.51	.777	1.18	0.72	1.93	.503	2.44%
Race									
White (Ref)	1.00	-	-	-	1.00	-	-	-	-
Black	0.83	0.72	0.95	.009	0.95	0.81	1.12	.572	-2.79%
Other Race	0.96	0.75	1.23	.759	1.06	0.81	1.38	.682	0.48%
Missing	0.89	0.61	1.30	.533	0.85	0.51	1.42	.533	-0.46%
Health insurance type									
Commercial (Ref)	1.00	-	-	-	1.00	-	-	-	-
Medicaid	0.80	0.68	0.95	.011	0.87	0.72	1.04	.133	-3.05%
Medicare	0.87	0.72	1.05	.154	0.87	0.71	1.07	.192	-0.37%
Uninsured	0.98	0.78	1.23	.860	1.00	0.79	1.27	.972	3.00%
Missing	0.82	0.54	1.26	.367	0.92	0.59	1.44	.714	1.24%
Education									
Some college or more (Ref)	1.00	-	-	-	1.00	-	-	-	-
Less than grade 12/GED	1.07	0.70	1.61	.763	0.86	0.71	1.04	.108	-6.81%
Grade 12 or GED	0.81	0.72	0.90	<.001	1.21	1.04	1.41	.015	5.47%
Missing	1.43	1.23	1.68	<.001	1.13	0.62	2.07	.687	1.45%
Has chronic condition	0.81	0.72	0.91	<.001	0.86	0.76	0.97	.013	-4.50%
Other tobacco products									
Uses cigar, pipe, or smokeless tobacco	1.07	0.87	1.31	.546	1.03	0.83	1.28	.776	2.34%
Uses other tobacco (e.g., hookah, e-cigarettes)	1.47	1.19	1.81	<.001	1.29	1.03	1.63	.030	5.06%
Smokes within 30 minutes of waking	0.65	0.57	0.74	<.001	0.66	0.58	0.76	<.001*	-8.91%

Hosmer-Lemeshow goodness-of-fit Chi Square(df=8)=9.99, p=0.265 for the adjusted model. OR=unadjusted odds ratio; aOR=adjusted odds ratio, 95%CI=95% confidence interval; Risk difference=difference in estimated outcome probability in adjusted model; Ref=reference group; * *p* value<Benjamini-Hochberg critical value.

Supplementary Table S3. Unadjusted (bivariate) and adjusted (multiple) logistic regression analyses of completing screening with potentially eligible participants on the phone (2,375 of 4,384, or 54.2%, of participants who smoked in the past 7 days at the recruitment call).

	Unadjusted				*Adjusted*				
Predictor	*OR*	*Low 95%CI*	*High 95%CI*	*p*	*aOR*	*Low 95%CI*	*High 95%CI*	*p*	*Risk Difference*
Wisconsin region									
Northeast (Ref)	1.00	-	-	-	1.00	-	-	-	-
Southeast	1.59	1.34	1.87	<.001	1.23	1.02	1.47	.027	9.36%
South	1.23	1.01	1.49	.043	1.14	0.93	1.39	.225	-1.14%
West	0.96	0.78	1.18	.246	1.01	0.82	1.25	.935	-7.62%
Age									
18 to 44 (Ref)	1.00	-	-	-	1.00	-	-	-	-
45 to 64	1.09	0.94	1.27	.240	1.01	0.87	1.19	.868	5.35%
65 to 90	0.64	0.53	0.76	<.001	0.65	0.53	0.80	<.001*	-11.80%
Sex									
Female (Ref)	1.00	-	-	-	1.00	-	-	-	
Male	0.84	0.75	0.95	.005	0.90	0.80	1.03	.118	-4.24%
Ethnicity									
Not Hispanic or Latino (Ref)	1.00	-	-	-	1.00	-	-	-	-
Hispanic or Latino	0.75	0.57	1.00	.053	0.69	0.50	0.94	.021	-6.93%
Missing	0.93	0.64	1.37	0.73	0.47	0.27	0.82	.008*	-6.48%
Race									
White (Ref)	1.00	-	-	-	1.00	-	-	-	
Black	2.54	2.18	2.97	<.001	2.32	1.95	2.76	<.001*	21.00%
Other Race	1.29	1.00	1.67	.053	1.33	1.01	1.77	.046	1.54%
Missing	1.51	1.02	2.24	.038	1.43	0.82	2.48	.203	3.11%
Health insurance type									
Commercial (Ref)	1.00	-	-	-	1.00	-	-	-	-
Medicaid	1.47	1.23	1.77	<.001	1.13	0.93	1.38	.231	9.67%
Medicare	0.97	0.79	1.18	.739	1.11	0.89	1.36	.343	-6.31%
Uninsured	1.03	0.81	1.32	.787	0.87	0.67	1.12	.277	-4.31%
Missing	1.00	0.65	1.55	.997	0.84	0.51	1.36	.469	-8.41%
Education									
Some college or more (Ref)	1.00	-	-	-	1.00	-	-	-	-
Less than grade 12/GED	0.72	0.60	0.85	<.001	0.61	0.50	0.74	<.001*	-0.62%
Grade 12 or GED	0.59	0.51	0.68	<.001	0.64	0.54	0.75	<.001*	-9.23%
Missing	1.01	0.65	1.58	.963	1.32	0.67	2.61	.429	6.44%
Has chronic condition	1.31	1.16	1.47	<.001	1.34	1.18	1.53	<.001*	6.83%
Other tobacco products									
Uses cigar, pipe, or smokeless tobacco	0.92	0.73	1.14	.435	0.91	0.72	1.16	.451	-1.31%
Uses other tobacco (e.g., hookah, e-cigarettes)	1.37	1.07	1.77	.014	1.31	1.00	1.72	.055	8.98%
Smokes within 30 minutes of waking	1.15	0.99	1.34	.078	1.17	1.00	1.37	.054	3.47%

Hosmer-Lemeshow goodness-of-fit Chi Square(df=8)=8.54, p=0.383 for the adjusted model. OR=unadjusted odds ratio; aOR=adjusted odds ratio, 95%CI=95% confidence interval; Risk difference=difference in estimated outcome probability in adjusted model; Ref=reference group; * *p* value<Benjamini-Hochberg critical value.

Supplementary Table S4. Unadjusted (bivariate) and adjusted (multiple) logistic regression analyses of meeting eligibility criteria at the study phone screen (1,744 of 2,375, or 73.4%, of participants screened met eligibility criteria).

	Unadjusted				*Adjusted*				
Predictor	*OR*	*Low 95%CI*	*High 95%CI*	*p*	*aOR*	*Low 95%CI*	*High 95%CI*	*p*	*Risk Difference*
Wisconsin region									
Northeast (Ref)	1.00	-	-	-	1.00	-	-	-	-
Southeast	0.79	0.61	1.04	.098	0.94	0.70	1.26	.659	-1.71%
South	0.89	0.65	1.23	0.49	0.93	0.66	1.30	.668	1.30%
West	0.82	0.58	1.15	.243	0.76	0.53	1.08	.122	-2.07%
Age									
18 to 44 (Ref)	1.00	-	-	-	1.00	-	-	-	-
45 to 64	1.09	0.87	1.36	.464	1.10	0.87	1.39	.424	3.08%
65 to 90	0.83	0.62	1.11	.206	0.91	0.66	1.27	.590	-4.49%
Sex									
Female (Ref)	1.00	-	-	-	1.00	-	-	-	
Male	1.24	1.03	1.50	.024	1.23	1.01	1.50	.039	3.68%
Ethnicity									
Not Hispanic or Latino (Ref)	1.00	-	-	-	1.00	-	-	-	-
Hispanic or Latino	1.14	0.70	1.86	.592	0.98	0.58	1.65	.944	2.49%
Missing	0.38	0.22	0.65	<.001	0.25	0.11	0.56	<.001*	-25.44%
Race									
White (Ref)	1.00	-	-	-	1.00	-	-	-	-
Black	0.64	0.52	0.78	<.001	0.63	0.50	0.79	<.001*	-8.43%
Other Race	1.09	0.72	1.66	.682	1.13	0.73	1.77	.577	4.01%
Missing	0.78	0.45	1.37	.391	1.09	0.48	2.46	.845	-9.55%
Health insurance type									
Commercial (Ref)	1.00	-	-	-	1.00	-	-	-	-
Medicaid	0.80	0.59	1.08	.137	0.79	0.58	1.09	.153	-0.71%
Medicare	0.71	0.51	1.00	.047	0.76	0.53	1.09	.134	-3.23%
Uninsured	0.85	0.57	1.27	.430	0.86	0.56	1.31	.475	0.97%
Missing	1.05	0.49	2.23	.900	1.13	0.50	2.55	.775	5.16%
Education									
Some college or more (Ref)	1.00	-	-	-	1.00	-	-	-	-
Less than grade 12/GED	1.00	0.77	1.30	.995	1.04	0.78	1.38	.803	3.29%
Grade 12 or GED	0.79	0.64	0.98	.034	0.79	0.62	1.00	.047	-3.35%
Unknown	0.61	0.34	1.09	.094	1.37	0.50	3.78	.540	-11.08%
Has chronic condition	1.06	0.89	1.28	.504	1.07	0.88	1.30	.480	1.00%
Other tobacco product use									
Uses cigar, pipe, or smokeless tobacco	1.02	0.72	1.44	.919	0.92	0.64	1.32	.638	-0.28%
Uses other tobacco (e.g., hookah, e-cigarettes)	0.88	0.62	1.26	.493	0.88	0.60	1.28	.491	-2.33%
Smokes within 30 minutes of waking	1.95	1.56	2.44	<.001	1.88	1.49	2.36	<.001*	13.13%

Hosmer-Lemeshow goodness-of-fit Chi Square(df=8)=8.60, p=0.377 for the adjusted model. OR=unadjusted odds ratio; aOR=adjusted odds ratio, 95%CI=95% confidence interval; Risk difference=difference in estimated outcome probability in adjusted model; Ref=reference group; * *p* value<Benjamini-Hochberg critical value.

Supplementary Table S5. Unadjusted (bivariate) and adjusted (multiple) logistic regression analyses of exclusion due to insufficient smoking out of all clients who were screened and not excluded due to pregnancy (476 of 2,357, or 20.2%, of participants screened were excluded due to insufficient smoking in the week before screening).

	Unadjusted				*Adjusted*				
Predictor	*OR*	*Low 95%CI*	*High 95%CI*	*p*	*aOR*	*Low 95%CI*	*High 95%CI*	*p*	*Risk Difference*
Wisconsin region									
Northeast (Ref)	1.00	-	-	-	1.00	-	-	-	-
Southeast	1.28	0.94	1.73	.115	0.98	0.70	1.36	.892	1.86%
South	1.22	0.85	1.74	.285	1.14	0.79	1.66	.479	0.57%
West	1.04	0.70	1.54	.836	1.17	0.78	1.76	.439	-0.87%
Age									
18 to 44 (Ref)	1.00	-	-	-	1.00	-	-	-	-
45 to 64	1.06	0.83	1.36	.641	1.08	0.83	1.41	.585	-0.29%
65 to 90	1.19	0.86	1.64	.295	1.17	0.81	1.70	.409	1.93%
Sex									
Female (Ref)	1.00	-	-	-	1.00	-	-	-	-
Male	0.83	0.68	1.03	.084	0.85	0.68	1.05	.128	-2.24%
Ethnicity									
Not Hispanic or Latino (Ref)	1.00	-	-	-	1.00	-	-	-	-
Hispanic or Latino	0.94	0.55	1.61	.814	1.13	0.64	2.01	.678	-0.36%
Missing	3.06	1.78	5.25	<.001	3.92	1.72	8.97	.001*	24.21%
Race									
White (Ref)	1.00	-	-	-	1.00	-	-	-	-
Black	1.99	1.60	2.47	<.001	2.03	1.57	2.62	<.001*	10.74%
Other Race	1.15	0.73	1.81	.560	1.08	0.66	1.76	.755	-0.96%
Missing	1.62	0.89	2.95	.112	1.06	0.44	2.56	.904	10.69%
Health insurance type									
Commercial (Ref)	1.00	-	-	-	1.00	-	-	-	-
Medicaid	1.53	1.07	2.17	.018	1.48	1.02	2.14	.037	1.91%
Medicare	1.54	1.04	2.26	.030	1.44	0.95	2.17	.084	1.51%
Uninsured	1.38	0.87	2.18	.171	1.34	0.83	2.17	.235	-0.59%
Missing	1.39	0.62	3.08	.422	1.15	0.48	2.74	.749	-1.80%
Education									
Some college or more (Ref)	1.00	-	-	-	1.00	-	-	-	-
Less than grade 12/GED	1.06	0.79	1.41	.696	1.00	0.73	1.37	.986	-1.63%
Grade 12 or GED	1.22	0.96	1.54	.108	1.29	0.99	1.68	.062	1.68%
Missing	1.99	1.09	3.63	.025	0.82	0.28	2.35	.707	12.88%
Has chronic condition	0.92	0.75	1.13	.427	0.90	0.72	1.12	.332	-1.02%
Other tobacco products									
Uses cigar, pipe, or smokeless tobacco	1.13	0.78	1.63	.524	1.27	0.86	1.87	.223	2.72%
Uses other tobacco (e.g., hookah, e-cigarettes)	1.22	0.84	1.79	.300	1.21	0.81	1.83	.354	3.45%
Smokes within 30 minutes of waking	0.51	0.40	0.64	<.001	0.54	0.42	0.69	<.001*	-10.73%

Hosmer-Lemeshow goodness-of-fit Chi Square(df=8)=2.64, p=0.955 for the adjusted model. OR=unadjusted odds ratio; aOR=adjusted odds ratio, 95%CI=95% confidence interval; Risk difference=difference in estimated outcome probability in adjusted model; Ref=reference group; * *p* value<Benjamini-Hochberg critical value.

Supplementary Table S6. Unadjusted (bivariate) and adjusted (multiple) logistic regression analyses of exclusion due to unwillingness to set a quit date within the next 2 weeks out of all clients who met other eligibility criteria (137 of 1,881, or 7.3%, of participants screened were excluded for this reason).

	Unadjusted				*Adjusted*				
Predictor	*OR*	*Low 95%CI*	*High 95%CI*	*p*	*aOR*	*Low 95%CI*	*High 95%CI*	*p*	*Risk Difference*
Wisconsin region									
Northeast (Ref)	1.00	-	-	-	1.00	-	-	-	-
Southeast	1.33	0.77	2.29	.307	1.48	0.84	2.62	.179	0.44%
South	0.96	0.49	1.88	.904	0.99	0.50	1.95	.964	-2.33%
West	1.93	1.03	3.61	.040	1.82	0.96	3.43	.066	3.53%
Age									
18 to 44 (Ref)	1.00	-	-	-	1.00	-	-	-	-
45 to 64	0.99	0.64	1.54	.966	0.93	0.59	1.48	.767	-1.56%
65 to 90	1.78	1.06	3.01	.030	1.44	0.79	2.64	.233	4.89%
Sex									
Female (Ref)	1.00	-	-	-	1.00	-	-	-	-
Male	0.90	0.63	1.29	.571	0.94	0.65	1.36	.732	-0.71%
Ethnicity									
Not Hispanic or Latino (Ref)	1.00	-	-	-	1.00	-	-	-	-
Hispanic or Latino	0.53	0.17	1.71	.287	0.72	0.21	2.44	.596	-3.64%
Missing	1.30	0.39	4.32	.670	2.90	0.60	13.91	.186	4.81%
Race									
White (Ref)	1.00	-	-	-	1.00	-	-	-	-
Black	0.74	0.49	1.12	.154	0.73	0.46	1.16	.183	-1.69%
Other Race	0.31	.10	0.99	.048	0.34	0.10	1.13	.077	-5.37%
Missing	0.50	.012	2.07	.334	0.39	0.06	2.79	.351	-1.94%
Health insurance type									
Commercial (Ref)	1.00	-	-	-	1.00	-	-	-	-
Medicaid	0.67	0.41	1.12	.126	0.76	0.44	1.30	.312	-2.23%
Medicare	1.03	0.59	1.79	.916	0.89	0.49	1.62	.713	2.67%
Uninsured	0.83	0.41	1.66	.590	0.88	0.42	1.82	.727	0.20%
Missing	0.28	0.04	2.15	.222	0.37	0.05	2.92	.345	-4.60%
Education									
Some college or more (Ref)	1.00	-	-	-	1.00	-	-	-	-
Less than grade 12/GED	0.82	0.49	1.39	.462	0.78	0.44	1.36	.371	-2.32%
Grade 12 or GED	1.34	0.88	1.96	.108	1.08	0.69	1.68	.741	2.20%
Missing	0.77	.18	3.32	.727	0.81	0.10	6.63	.842	-0.86%
Has chronic condition	1.10	0.78	1.56	.594	1.08	0.75	1.56	.670	0.63%
Other tobacco products									
Uses cigar, pipe, or smokeless tobacco	0.59	0.24	1.31	.179	0.56	0. 24	1.34	.196	-3.68%
Uses other tobacco (e.g., hookah, e-cigarettes)	0.81	0.67	1.79	.599	0.91	0.40	2.05	.817	-1.56%
Smokes within 30 minutes of waking	0.58	0.38	0.88	.011	0.59	0.39	0.91	.017	-4.22%

Hosmer-Lemeshow goodness-of-fit Chi Square(df=8)=5.53, p=0.699 for the adjusted model. OR=unadjusted odds ratio; aOR=adjusted odds ratio, 95%CI=95% confidence interval; Risk difference=difference in estimated outcome probability in adjusted model; Ref=reference group; * *p* value<Benjamini-Hochberg critical value.

Supplementary Table S7. Unadjusted (bivariate) and adjusted (multiple) logistic regression analyses of trial enrollment among all those who met screening criteria (1,316 of 1,744, or 75.5%, of eligible participants enrolled and were randomized).

	Unadjusted				*Adjusted*				
Predictor	*OR*	*Low 95%CI*	*High 95%CI*	*p*	*aOR*	*Low 95%CI*	*High 95%CI*	*p*	*Risk Difference*
Wisconsin region									
Northeast (Ref)	1.00	-	-	-	1.00	-	-	-	-
Southeast	0.83	0.60	1.13	.235	0.83	0.59	1.16	.277	-3.98%
South	1.08	0.74	1.59	.680	1.10	0.74	1.64	.632	3.67%
West	0.99	0.66	1.49	.963	1.01	0.66	1.54	.953	1.70%
Age									
18 to 44 (Ref)	1.00	-	-	-	1.00	-	-	-	-
45 to 64	1.10	0.85	1.44	.474	1.14	0.86	1.51	.350	2.82%
65 to 90	0.85	0.60	1.27	.364	1.04	0.70	1.56	.845	-4.58%
Sex									
Female (Ref)	1.00	-	-	-	1.00	-	-	-	-
Male	1.15	0.92	1.44	.223	1.18	0.94	1.50	.157	2.44%
Ethnicity									
Not Hispanic or Latino (Ref)	1.00	-	-	-	1.00	-	-	-	-
Hispanic or Latino	0.94	0.55	1.63	.835	0.86	0.47	1.56	.613	-1.12%
Missing	0.52	0.25	1.12	.095	0.12	0.03	0.45	.002*	-24.09%
Race									
White (Ref)	1.00	-	-	-	1.00	-	-	-	-
Black	0.93	0.73	1.19	.570	1.04	0.78	1.38	.792	-1.27%
Other Race	1.20	0.74	1.94	.451	1.30	0.77	2.19	.331	3.96%
Missing	1.51	0.69	3.27	.302	1.30	0.45	3.80	.626	0.96%
Health insurance type									
Commercial (Ref)	1.00	-	-	-	1.00	-	-	-	-
Medicaid	1.26	0.90	1.76	.178	1.08	0.76	1.54	.661	5.54%
Medicare	0.84	0.85	1.22	.364	0.85	0.57	1.27	.432	-6.82%
Uninsured	1.11	0.71	1.76	.645	0.96	0.59	1.53	.848	-0.14%
Missing	1.49	0.62	3.58	.373	1.34	0.51	3.50	.551	3.51%
Education									
Some College or More (Ref)	1.00	-	-	-	1.00	-	-	-	-
Less than Grade 12 or GED	0.67	0.49	0.92	.012	0.70	0.50	0.98	.038	-0.10%
Grade 12 or GED	0.56	0.43	0.73	<.001	0.60	0.45	0.79	<.001*	-7.90%
Missing	1.42	0.54	3.74	.478	4.86	0.91	25.92	.064	7.55%
Has chronic condition	1.17	0.94	1.45	.166	1.23	0.97	1.55	.480	3.38%
Other tobacco products									
Uses cigar, pipe, or smokeless tobacco	1.28	0.82	2.00	.272	1.18	0.74	1.88	.480	5.29%
Uses other tobacco (e.g., hookah, e-cigarettes))	1.35	0.84	2.17	.223	1.29	0.78	2.15	.322	6.15%
Smokes within 30 minutes of waking	1.23	0.92	1.66	.165	1.20	0.89	1.63	.231	3.50%

Hosmer-Lemeshow goodness-of-fit Chi Square(df=8)=11.34, p=0.183 for the adjusted model. OR=unadjusted odds ratio; aOR=adjusted odds ratio, 95%CI=95% confidence interval; Risk difference=difference in estimated outcome probability in adjusted model; Ref=reference group; * *p* value<Benjamini-Hochberg critical value.

Supplementary Table S8. Unadjusted (bivariate) and adjusted (multiple) logistic regression analyses of receiving NRT among all enrolled and randomized trial participants (1,060 of 1,316 enrolled, or 80.5%, of participants received NRT).

	Unadjusted				*Adjusted*				
Predictor	*OR*	*Low 95%CI*	*High 95%CI*	*p*	*aOR*	*Low 95%CI*	*High 95%CI*	*p*	*Risk Difference*
Age									
18 to 44 (Ref)	1.00	-	-	-	1.00	-	-	-	-
45 to 64	1.13	0.82	1.55	.473	1.14	0.78	1.66	.502	-1.04%
65 to 90	1.59	0.98	2.58	1.59	1.82	0.98	3.35	.056	6.33%
Sex									
Female (Ref)	1.00	-	-	-	1.00	-	-	-	-
Male	0.95	0.72	1.25	.705	0.83	0.60	1.14	.244	-2.37%
Ethnicity									
Not Hispanic or Latino (Ref)	1.00	-	-	-	1.00	-	-	-	-
Hispanic or Latino	1.75	0.74	4.16	.205	1.68	0.66	4.27	.280	4.45%
Race									
White (Ref)	1.00	-	-	-	1.00	-	-	-	-
Black	1.22	0.88	1.69	.229	1.06	0.71	1.58	.777	3.11%
Other race	0.92	0.55	1.56	.77	0.66	0.37	1.18	.164	-4.89%
Health insurance type									
Commercial (Ref)	1.00	-	-	-	1.00	-	-	-	-
Medicaid	0.87	0.56	1.36	.543	0.60	0.64	1.06	0.80	-1.82%
Medicare	0.94	0.56	1.59	.824	0.70	0.38	1.32	.275	1.55%
Uninsured	0.70	0.40	1.24	.221	0.60	0.30	1.20	.147	-2.98%
Education									
Some college or more (Ref)	1.00	-	-	-	1.00	-	-	-	-
Less than grade 12/GED	0.84	0.58	1.21	.338	0.75	0.48	1.19	.225	-1.11%
Grade 12 or GED	0.93	0.68	1.28	.655	0.86	0.58	1.27	.436	0.39%
Has chronic condition	1.10	0.83	1.44	.518	0.97	0.71	1.34	.871	0.60%
Other tobacco products									
Uses cigar, pipe, or smokeless tobacco	1.50	0.73	3.07	.269	2.48	1.04	5.91	.041	8.41%
Uses other tobacco (e.g., hookah, e-cigarettes)	0.87	0.46	1.67	.683	0.81	0.40	1.65	.562	-3.20%
Smokes within 30 minutes of waking	0.98	0.68	1.40	.891	1.08	0.73	1.58	.707	0.42%
Annual household income									
At or above median	1.00	-	-	-	1.00	-	-	-	-
Below median	1.16	0.86	1.56	.326	1.09	0.76	1.56	.658	1.07%
Missing	1.10	0.74	1.65	.634	1.13	0.70	1.83	.628	0.66%
ADI percentile	1.01	1.00	1.01	.054	1.01	1.00	1.02	.011	4.66%**

Hosmer-Lemeshow goodness-of-fit Chi Square(df=8)=2.58, p=0.958 for the adjusted model. OR=unadjusted odds ratio; aOR=adjusted odds ratio, 95%CI=95% confidence interval; Risk difference=difference in estimated outcome probability in adjusted model; Ref=reference group; * *p* value<Benjamini-Hochberg critical value.
** Risk Difference is for those at or above vs below the median national ADI 85th percentile. National ADI percentile was correlated 0.521 with estimated probability of receiving NRT.

Supplementary Table S9. Unadjusted (bivariate) and adjusted (multiple) logistic regression analyses of any self-reported use of NRT at the 12-week post-quit follow-up call among participants who completed the 12-week follow-up (966 of 1,173, or 82.4%, who completed the 12-week follow-up reported any use of NRT [143, or 10.9% of the 1,316 randomized trial enrollees did not complete the 12-week follow-up]).

	Unadjusted				*Adjusted*				
Predictor	*OR*	*Low 95%CI*	*High 95%CI*	*p*	*aOR*	*Low 95%CI*	*High 95%CI*	*p*	*Risk Difference*
Age									
18 to 44 (Ref)	1.00	-	-	-	1.00	-	-	-	-
45 to 64	1.01	0.70	1.47	.939	1.08	0.71	1.63	.721	1.10%
65 to 90	0.98	0.59	1.62	.939	1.09	0.61	1.97	.768	-0.94%
Sex									
Female (Ref)	1.00	-	-	-	1.00	-	-	-	-
Male	0.94	0.70	1.28	.710	0.97	0.69	1.36	.849	-0.75%
Ethnicity									
Not Hispanic or Latino (Ref)	1.00	-	-	-	1.00	-	-	-	-
Hispanic or Latino	0.83	0.39	1.75	.622	0.73	0.33	1.66	.457	-5.25%
Race									
White (Ref)	1.00	-	-	-	1.00	-	-	-	-
Black	0.95	0.68	1.33	.758	1.05	0.69	1.58	.828	0.62%
Other race	0.88	0.50	1.54	.650	0.96	0.51	1.82	.910	-1.72%
Health insurance type									
Commercial (Ref)	1.00	-	-	-	1.00	-	-	-	-
Medicaid	0.97	0.59	1.60	.905	0.86	0.48	1.54	.613	1.92%
Medicare	0.78	0.44	1.38	.393	0.77	0.51	1.18	.231	-4.19%
Uninsured	1.04	0.53	2.04	.899	0.88	0.42	1.84	.733	0.64%
Education									
Some college or more (Ref)	1.00	-	-	-	1.00	-	-	-	-
Less than grade 12/GED	0.74	0.50	1.13	.166	0.83	0.51	1.36	.465	-0.26%
Grade 12 or GED	0.80	0.56	1.14	.214	0.77	0.51	1.18	.231	-2.82%
Has chronic condition	1.10	0.81	1.49	.541	1.05	0.75	1.47	.796	0.49%
Other tobacco products									
Uses cigar, pipe, or smokeless tobacco	1.09	0.56	2.13	.799	1.40	0.64	3.04	.398	4.09%
Uses other tobacco (e.g., hookah, e-cigarettes)	0.72	0.38	1.36	.308	0.84	0.40	1.73	.629	-2.33%
Smokes within 30 minutes of waking	1.12	0.77	1.62	.565	1.14	0.77	1.69	.508	1.84%
Annual household income									
At or above median	1.00	-	-	-	1.00	-	-	-	-
Below median	1.04	0.75	1.44	.829	1.07	0.74	1.57	.710	0.58%
Missing	1.02	0.66	1.60	.918	1.18	0.71	1.96	.531	1.27%
ADI percentile	1.00	0.99	1.01	.498	1.00	0.99	1.01	.679	-0.72%**

Hosmer-Lemeshow goodness-of-fit Chi Square(df=8)=8.28, p=0.407 for the adjusted model. OR=unadjusted odds ratio; aOR=adjusted odds ratio, 95%CI=95% confidence interval; Risk difference=difference in estimated outcome probability in adjusted model; Ref=reference group; * *p* value<Benjamini-Hochberg critical value.
** Risk Difference is for those at or above vs below the median national ADI 85th percentile. National ADI percentile was correlated -0.160 with estimated probability of reporting any NRT use at the 12 week follow-up.

Supplementary Table S10. Unadjusted (bivariate) and adjusted (multiple) logistic regression analyses of any self-reported use of NRT at the 26-week post-quit follow-up call among participants who completed the 26-week follow-up (555 of 1,133, or 49.0%, who completed the 26-week follow-up reported any use of NRT [183, or 13.9% of the 1,316 randomized trial enrollees did not complete the 12-week follow-up]).

	Unadjusted				*Adjusted*				
Predictor	*OR*	*Low 95%CI*	*High 95%CI*	*p*	*aOR*	*Low 95%CI*	*High 95%CI*	*p*	*Risk Difference*
Age									
18 to 44 (Ref)	1.00	-	-	-	1.00	-	-	-	-
45 to 64	1.29	0.97	1.72	.083	1.29	0.94	1.78	.115	-0.59%
65 to 90	1.99	1.33	2.97	<.001	2.42	1.51	3.86	<.001*	12.69%
Sex									
Female (Ref)	1.00	-	-	-	1.00	-	-	-	-
Male	0.99	0.78	1.26	.951	1.13	0.87	1.47	.369	0.51%
Ethnicity									
Not Hispanic or Latino (Ref)	1.00	-	-	-	1.00	-	-	-	-
Hispanic or Latino	0.63	0.34	1.19	.155	0.68	0.34	1.38	.287	-10.96%
Race									
White (Ref)	1.00	-	-	-	1.00	-	-	-	-
Black	1.48	1.13	1.93	.004	1.56	1.14	2.14	.005*	11.70%
Other race	0.89	0.56	1.40	.603	0.93	0.56	1.55	.782	-7.43%
Health insurance type									
Commercial (Ref)	1.00	-	-	-	1.00	-	-	-	-
Medicaid	1.22	0.84	1.78	.299	1.04	0.67	1.62	.861	3.26%
Medicare	1.09	0.70	1.70	.695	0.86	0.53	1.11	.555	-2.03%
Uninsured	1.14	0.69	1.88	.621	0.91	0.51	1.60	.734	-3.00%
Education									
Some college or more (Ref)	1.00	-	-	-	1.00	-	-	-	-
Less than grade 12/GED	0.88	0.64	1.20	.413	0.77	0.53	1.42	.702	-0.27%
Grade 12 or GED	0.77	0.59	1.00	.050	0.74	0.54	1.02	.067	-5.04%
Has chronic condition	1.01	0.80	1.27	.966	0.91	0.71	1.19	.500	-0.72%
Other tobacco products									
Uses cigar, pipe, or smokeless tobacco	0.79	0.48	1.31	.356	0.92	0.53	1.58	.749	-2.10%
Uses other tobacco (e.g., hookah, e-cigarettes)	1.05	0.61	1.81	0.851	0.92	0.51	1.68	.790	-3.26%
Smokes within 30 minutes of waking	1.07	0.80	1.42	.666	1.20	0.88	1.64	.245	1.96%
Annual household income									
At or above median	1.00	-	-	-	1.00	-	-	-	-
Below median	1.19	0.92	1.53	.183	1.06	0.79	1.62	.861	2.95%
Missing	1.03	0.72	1.46	.891	0.99	0.67	1.46	.947	0.28%
ADI percentile	1.01	1.00	1.01	.072	1.00	1.00	1.01	.352	5.85%**

Hosmer-Lemeshow goodness-of-fit Chi Square(df=8)=8.20, p=0.414 for the adjusted model. OR=unadjusted odds ratio; aOR=adjusted odds ratio, 95%CI=95% confidence interval; Risk difference=difference in estimated outcome probability in adjusted model; Ref=reference group; * *p* value<Benjamini-Hochberg critical value.
** Risk Difference is for those at or above vs below the median national ADI 85th percentile. National ADI percentile was correlated 0.315 with estimated probability of reporting any NRT use at the 26 week follow-up.

Supplementary Table S11. Unadjusted (bivariate) and adjusted (multiple) logistic regression analyses of self-reported abstinence from smoking in the past 7 days at the 12-week post-quit follow-up call among all randomized enrollees (336 of 1,316, or 25.5%, of enrollees reported no smoking in the past 7 days).

	Unadjusted				*Adjusted*				
Predictor	*OR*	*Low 95%CI*	*High 95%CI*	*p*	*aOR*	*Low 95%CI*	*High 95%CI*	*p*	*Risk Difference*
Age									
18 to 44 (Ref)	1.00	-	-	-	1.00	-	-	-	-
45 to 64	1.17	0.86	1.59	.324	1.27	0.90	1.80	.174	1.23%
65 to 90	1.19	0.78	1.80	.425	1.41	0.86	2.30	.172	2.04%
Sex									
Female (Ref)	1.00	-	-	-	1.00	-	-	-	-
Male	1.11	0.87	1.43	.398	1.01	0.76	1.34	.946	1.86%
Ethnicity									
Not Hispanic or Latino (Ref)	1.00	-	-	-	1.00	-	-	-	-
Hispanic or Latino	2.26	1.26	4.03	.006	2.89	1.49	5.63	.002*	17.96%
Race									
White (Ref)	1.00	-	-	-	1.00	-	-	-	-
Black	1.09	0.82	1.44	.553	1.03	0.74	1.45	.846	1.66%
Other race	0.92	0.55	1.51	.730	0.73	0.41	1.30	.284	-1.02%
Health insurance type									
Commercial (Ref)	1.00	-	-	-	1.00	-	-	-	-
Medicaid	1.14	0.76	1.70	.528	1.34	0.84	2.14	.218	1.20%
Medicare	1.24	0.78	1.97	.367	1.14	0.68	1.90	.616	1.02%
Uninsured	1.09	0.64	1.86	.756	1.28	0.70	2.33	.417	-1.13%
Education									
Some college or more (Ref)	1.00	-	-	-	1.00	-	-	-	-
Less than grade 12/GED	1.29	0.92	1.81	.146	1.50	1.01	2.24	.044	3.21%
Grade 12 or GED	1.27	0.95	1.70	.105	1.48	1.05	2.10	.025	2.13%
Has chronic condition	0.85	0.66	1.09	.191	0.70	0.53	0.92	.011	-4.90%
Other tobacco products									
Uses cigar, pipe, or smokeless tobacco	1.60	0.96	2.65	.071	1.64	0.94	2.83	.080	10.27%
Uses other tobacco (e.g., hookah, e-cigarettes)	1.07	0.61	1.89	.807	1.19	0.63	2.24	.585	3.56%
Smokes within 30 minutes of waking	0.68	0.50	0.91	.010	0.66	0.48	0.91	.011	-8.77%
Annual household income									
At or above median	1.00	-	-	-	1.00	-	-	-	-
Below median	1.33	1.02	1.75	.039	1.30	0.95	1.78	.104	4.32%
Missing	1.23	0.85	1.78	.274	1.13	0.74	1.72	.569	0.70%
ADI percentile	1.00[g]	0.99	1.01	.776	1.00	0.99	1.00	.419	0.34%**

Hosmer-Lemeshow goodness-of-fit Chi Square(df=8)=2.62, p=0.956 for the adjusted model. OR=unadjusted odds ratio; aOR=adjusted odds ratio, 95%CI=95% confidence interval; Risk difference=difference in estimated outcome probability in adjusted model; Ref=reference group; * *p* value<Benjamini-Hochberg critical value.
** Risk Difference is for those at or above vs below the median national ADI 85th percentile. National ADI percentile was correlated 0.001 with estimated probability of reporting no smoking in the past 7 days at the 12 week follow-up.

Supplementary Table S12. Unadjusted (bivariate) and adjusted (multiple) logistic regression analyses of salivary cotinine-confirmed abstinence from smoking in the past 7 days at the 26-week post-quit follow-up call among all randomized enrollees (162 of 1,316, or 12.3%, of enrollees claimed abstinence and returned saliva samples at the 26-week follow-up).

	Unadjusted				*Adjusted*				
Predictor	*OR*	*Low 95%CI*	*High 95%CI*	*p*	*aOR*	*Low 95%CI*	*High 95%CI*	*p*	*Risk Difference*
Age									
18 to 44 (Ref)	1.00	-	-	-	1.00	-	-	-	-
45 to 64	1.26	0.83	1.92	.275	1.44	0.89	2.33	.141	2.49%
65 to 90	1.24	0.71	2.18	.453	1.21	0.61	2.42	.586	-1.05%
Sex									
Female (Ref)	1.00	-	-	-	1.00	-	-	-	-
Male	0.58	0.41	0.82	.002	0.49	0.33	0.73	<.001*	-6.20%
Ethnicity									
Not Hispanic or Latino (Ref)	1.00	-	-	-	1.00	-	-	-	-
Hispanic or Latino	3.34	1.78	6.28	<.001	5.73	2.68	12.21	<.001*	21.25%
Race									
White (Ref)	1.00	-	-	-	1.00	-	-	-	-
Black	1.55	1.09	2.22	.016	1.34	0.86	2.09	.191	4.73%
Other race	1.09	0.56	2.12	.802	0.60	0.27	1.32	.207	0.47%
Health insurance type									
Commercial (Ref)	1.00	-	-	-	1.00	-	-	-	-
Medicaid	1.09	0.62	1.81	.836	1.13	0.59	2.16	.711	-0.41%
Medicare	1.40	0.76	2.56	.280	1.28	0.64	2.57	.482	1.35%
Uninsured	1.04	0.51	2.12	.923	1.43	0.64	3.21	.380	-0.41%
Education									
Some college or more (Ref)	1.00	-	-	-	1.00	-	-	-	-
Less than grade 12/GED	1.29	0.82	2.03	.267	1.24	0.73	2.11	.434	2.02%
Grade 12 or GED	1.24	0.84	1.83	.281	1.28	0.80	2.05	.307	-0.14%
Has chronic condition	0.78	0.56	1.08	.137	0.62	0.42	0.90	.012	-3.51%
Other tobacco products									
Uses cigar, pipe, or smokeless tobacco	1.09	0.53	2.25	.818	1.67	0.78	3.61	.189	1.96%
Uses other tobacco (e.g., hookah, e-cigarettes)	0.59	0.23	1.50	.265	0.81	0.30	2.19	.676	-3.09%
Smokes within 30 minutes of waking	0.86	0.58	1.29	.476	0.89	0.58	1.39	.613	-1.56%
Annual household income									
At or above median	1.00	-	-	-	1.00	-	-	-	-
Below median	1.27	0.88	1.81	.201	1.18	0.77	1.82	.452	1.79%
Missing	1.19	0.73	1.94	.496	1.21	0.69	2.12	.505	1.42%
ADI percentile	1.01	1.00	1.02	.048	1.00	0.99	1.01	.578	3.76%**

Hosmer-Lemeshow goodness-of-fit Chi Square(df=8)=4.72, p=0.787 for the adjusted model. OR=unadjusted odds ratio; aOR=adjusted odds ratio, 95%CI=95% confidence interval; Risk difference=difference in estimated outcome probability in adjusted model; Ref=reference group; * *p* value<Benjamini-Hochberg critical value.
** Risk Difference is for those at or above vs below the median national ADI 85th percentile. National ADI percentile was correlated 0.245 with estimated probability of biochemically verified past-7-day abstinence at the 26 week follow-up.

Supplementary Figure S1. Secondary analyses of differential flow through parent trial milestones among 14125 prior Wisconsin Tobacco Quit Line participants identified for recruitment between June 7, 2018 and January 25, 2023.

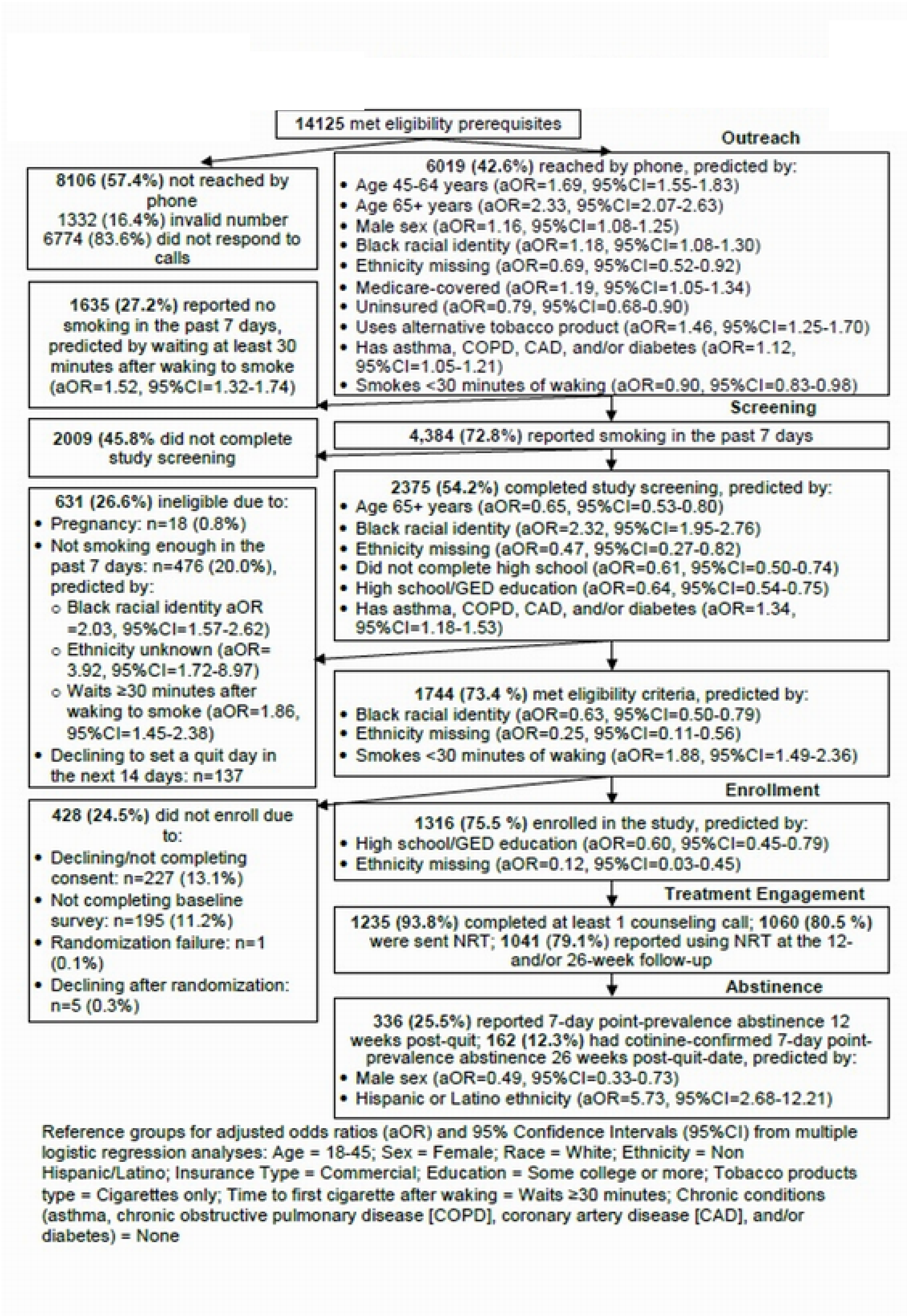

Supplement: Supplementary file 1 [file TPC-12-63-s001.pdf]
